# ASSOCIATION OF ASSISTED LIVING HOMES PREPAREDNESS AND RESPONSES TO THE COVID-19 PANDEMIC WITH RESIDENT LONELINESS

**DOI:** 10.1093/geroni/igad104.1629

**Published:** 2023-12-21

**Authors:** Rashmi Devkota, Colleen Maxwell, Hana Dampf, Shovana Shrestha, Matthias Hoben

**Affiliations:** University of Alberta, Edmonton, Alberta, Canada; University of Waterloo, Waterloo, Ontario, Canada; University of Alberta, Edmonton, Alberta, Canada; University of Alberta, Edmonton, Alberta, Canada; York University, Toronto, Ontario, Canada

## Abstract

Loneliness has been an ongoing problem among Assisted living (AL) residents and has worsened during the COVID-19 pandemic. Loneliness is associated with an increased risk of mental and physical illness, cognitive decline, suicidal behavior, and all-cause mortality. However, the impact of the pandemic on AL residents’ loneliness is poorly understood. We surveyed 42 AL homes in Alberta to understand whether resident characteristics and homes’ pandemic preparedness and response to the pandemic were associated with resident loneliness. The surveys reflected pandemic waves 1 (Mar-Jul 2020) and 2 (Nov 2020-Feb 2021) and were linked to Resident Assessment Instrument (RAI) records of residents who lived in these homes during these periods. Using generalized estimating equation models, we assessed whether resident characteristics, residents’ social relationships, and home preparedness for and responses to the pandemic were associated with resident loneliness (measured as present or absent based on RAI items). Our sample included 1,828 residents (wave 1: 890, wave 2: 938). Almost 13% of the residents reported loneliness (wave 1: 11.2%, wave 2: 14.2%). Depressive symptoms (odds ratio [OR]=2.73, 95% CI, 1.9-3.9) daily/excruciating pain (OR=1.97, 95% CI, 1.4-2.8), and cognitive impairment (OR=0.38, 95% CI, 0.2-0.6) were significantly associated with loneliness. Caregiver availability, hours of caregiver help, and home preparedness were not associated with loneliness, but more communication with caregivers (OR=2.19, 95% CI, 1.1-4.2) and same or improved staff morale (OR=0.61, 95% CI, 0.4-0.9) were. Improving staff morale and communication with caregivers is crucial in addressing resident loneliness.

